# Telehealth services utilization among middle-aged and older informal caregivers: evidence from national-level data in the United States

**DOI:** 10.3389/fpubh.2026.1859507

**Published:** 2026-06-16

**Authors:** Asos Mahmood, Nikhil A. Ahuja, Coree Entwistle, Satish Kedia, Courtney Sievers, Cyril F. Chang

**Affiliations:** 1 Center for Health System Improvement, College of Medicine, University of Tennessee Health Science Center, Memphis, TN, United States; 2 Department of Medicine-General Internal Medicine, College of Medicine, University of Tennessee Health Science Center, Memphis, TN, United States; 3 Tennessee Population Health Consortium, University of Tennessee Health Science Center, Memphis, TN, United States; 4 Department of Public Health Sciences, Slippery Rock University of Pennsylvania, Slippery Rock, PA, United States; 5 Division of Social and Behavioral Sciences, School of Public Health, University of Memphis, Memphis, TN, United States; 6 Premise Health, Brentwood, TN, United States; 7 Fogelman College of Business and Economics, University of Memphis, Memphis, TN, United States

**Keywords:** caregiving, COVID-19, health services utilization, healthcare disparities, informal caregiver, middle-aged and older adults, telehealth

## Abstract

**Introduction:**

Telehealth emerged as a vital tool for healthcare delivery during the COVID-19 pandemic. However, its utilization among middle-aged and older informal caregivers remains insufficiently explored in the U.S. This study examines the relationship between informal caregiving status and telehealth service utilization, including the modes of use, motivators, and barriers experienced during the pandemic in the U.S.

**Methods:**

Data were derived from the Health Information National Trends Survey (HINTS6), a nationally representative, cross-sectional survey of U.S. adults (*n* = 3,354, ages ≥50 years). Multivariable multinomial logistic regression assessed the association between caregiving status and telehealth utilization, while Rao–Scott chi-square tests examined bivariate relationships.

**Results:**

Approximately 20% reported being informal caregivers. Compared to non-caregivers, caregivers were significantly more likely to have received (adjusted odds ratio [aOR] = 1.78; 95% CI: 1.31, 2.40) telehealth services during the pandemic. Caregivers more frequently cited convenience (66.4% vs. 56.2%; *p* = 0.036) and the ability to include family or other caregivers in appointments (30.8% vs. 19.0%; *p* = 0.037) as key motivators for telehealth use. Among those who were offered but who did not use telehealth, caregivers’ preference for in-person care was nearly universal (98.0% vs. 89.7%; *p* = 0.043), but privacy concerns trended lower (7.9% vs. 17.3%; *p* = 0.06).

**Conclusion:**

Informal caregivers exhibited higher rates of telehealth utilization and reported unique motivators and barriers. To enhance caregiver engagement and healthcare access, telehealth programs should optimize access and prioritize convenience, enable multi-participant visits, strengthen telehealth features that support care coordination. The findings may inform targeted telehealth policies and interventions for supporting this essential and vulnerable population.

## 1 Introduction

Telehealth refers to the use of digital communication technologies to deliver healthcare services, disseminate health information, and provide patient education (1–3). It has emerged as an effective tool to enhance healthcare access, reduce costs, improve patient satisfaction, and streamline healthcare delivery by making services more efficient and convenient. During the COVID-19 pandemic, telehealth experienced unprecedented growth, offering remote consultations, diagnoses, and treatment as alternatives to in-person care (1–6). This shift helped minimize the risk of viral exposure and infection (7, 8). In the United States (U.S.), telehealth utilization surged, driven primarily by the reduced availability of in-person services and facilitated in part by temporary federal and state policy waivers (9). However, access to telehealth remained limited for vulnerable populations, including older adults, individuals with chronic illnesses, and informal caregivers, who faced heightened risks and unique challenges in securing essential healthcare for themselves (10, 11).

Informal caregivers, unpaid individuals who provide essential care to family members, friends, or others with medical or functional needs, perform a wide range of tasks, from managing medical regimens to coordinating provider care and offering emotional support (12). These responsibilities often come at a significant personal cost, including emotional, physical, mental, and financial strain (10, 13). Specifically, older caregivers often navigate their own age-related health conditions in addition to caregiving responsibilities, increasing the importance of accessible healthcare modalities such as telehealth (14, 15). As of 2025, an estimated 63 million American adults (approximately 25%) served as informal caregivers, and more than half (55%) of them are 50 years of age or older (13). Despite their critical contributions to the healthcare system, supporting the system’s foundation and helping to reduce strain on already overburdened services, caregivers remain largely an underrecognized group (16–18). Meanwhile, demand for informal caregiving is expected to rise due to factors such as increased life expectancy, growing healthcare costs, workforce shortages in skilled nursing, and the desire of older adults to age in place (13, 19).

Given the intense demands placed on caregivers and their heightened vulnerability, exploring the role of telehealth in supporting this population during the pandemic is both timely and necessary. Prior research has shown that telehealth can enhance caregivers’ access to medical care, mental health support, and educational resources, while reducing travel burdens and offering more flexible scheduling (20). For example, telehealth is a viable tool to improve caregivers’ access to medical consultations, mental health support, and other educational resources, thereby improving health outcomes while alleviating burdens associated with caregiving responsibilities (21). These benefits have the potential to improve both caregiver and care recipient outcomes. Understanding how caregiving responsibilities influenced telehealth access and use during the COVID-19 pandemic is essential for developing targeted interventions that support caregivers and strengthen health system resilience.

Despite a growing body of literature on telehealth during the pandemic, few studies have focused specifically on middle-aged and older informal caregivers or the ways in which their caregiving roles shaped telehealth utilization (22, 23). The published research on telehealth service access and utilization during the COVID-19 pandemic in the U.S. has focused predominantly on disparities among the general adult population (24–30) or specific groups such as Medicare (2, 19, 31–33) and Medicaid (34) beneficiaries. Telehealth use disparities in other sociodemographic and particular patient groups, healthcare and community settings, and geographic regions in the U.S. have also been documented extensively (4, 7, 35–39). However, the intersection between informal caregiving status among those 50 years and older and telehealth access, particularly within the unique context of the COVID-19 pandemic, remains understudied (40). Furthermore, only a few studies have examined which factors influenced caregivers’ decisions to adopt or avoid telehealth, including motivations, logistical challenges, and perceived usefulness—areas critical for designing caregiver-centered interventions (41–44).

To address these gaps, we analyzed data from a nationally representative survey of U.S. adults (ages ≥50 years) who sought healthcare during the COVID-19 pandemic. Our primary aim was to investigate the association between informal caregiving status and telehealth service utilization. Additionally, we examined the modes of telehealth use, as well as key motivators and deterrents experienced by middle-aged and older informal caregivers during the pandemic. By identifying disparities in telehealth access and utilization affecting caregivers, this study seeks to inform policies and interventions to better support this vulnerable population, with implications for both ongoing and future public health preparedness.

## 2 Methods

### 2.1 Data sources, settings, and the analytic sample

This study analyzed secondary data from the Health Information National Trends Survey (HINTS), specifically the sixth iteration (HINTS6). HINTS is a national cross-sectional, population-representative survey of noninstitutionalized adults 18 years and older and has been administered routinely by the U.S. National Cancer Institute since 2003 (45–48). The survey has historically utilized a self-administered mailed questionnaire to collect data (49). However, for HINTS6, a multimode survey methodology was applied, and potential survey respondents were given two options to respond to the questionnaire: either through the traditional paper-and-pencil mode or electronically via the survey website (50).

**Figure 1:**
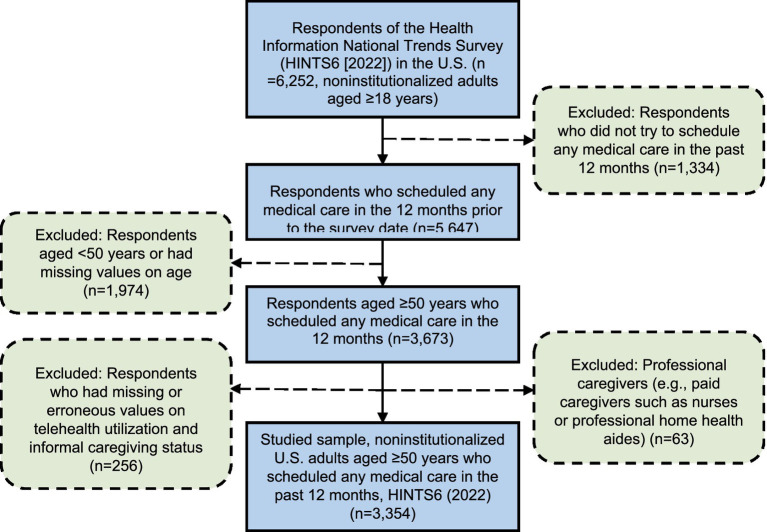
A flowchart illustrating the study’s inclusion and exclusion criteria.

HINTS6, fielded in 2022, comprised 6,252 respondents with an overall response rate of 28.1%. However, our analytical sample includes 3,354 middle-aged and older respondents (i.e., ages ≥ 50 years) who reported trying to schedule any medical care in the 12 months prior to the survey date. Figure 1 shows the study’s inclusion and exclusion criteria, while further details about HINTS design, methodology, data collection, and access are available at: https://hints.cancer.gov/ and discussed elsewhere (45, 51). Given that our study used publicly available, deidentified, secondary data, it was exempt from obtaining additional Institutional Review Board (IRB) approval and was not deemed human subjects research (52).

### 2.2 Telehealth service utilization

The primary outcome variable was “telehealth service utilization,” operationalized using two HINTS6 items. Participants were asked, “In the past 12 months, did you receive care from a doctor or health professional using telehealth?” and “In the past 12 months, were you offered the option to have a telehealth visit for any medical care you tried to schedule?” Based on the responses, we created a three-subcategory nominal variable pertaining to telehealth use. Respondents with an affirmative response to the first question were assigned “received” telehealth (including telehealth by video only, by phone call [voice only with no video], and both video and phone call). Respondents with a negative response to the first but affirmative response to the second question were assigned “offered but did not receive,” and respondents with a negative response to both questions were assigned “not offered nor received” telehealth. Survey respondents who did not try to schedule any medical care in the past 12 months were excluded from the study sample because they were not asked these questions (Figure 1).

### 2.3 Modes, motivators, and deterrents of telehealth use

For those who used telehealth services, we determined the following modes: “video only, audio only, and both video and audio.” Furthermore, motivators of telehealth use were assessed based on the survey question, “Why did you choose a telehealth visit(s) for yourself?,” with a series of options, “The health care provider recommended or required the visit use telehealth,” “I wanted advice about whether I needed in-person medical care,” “I wanted to avoid possible infection at the doctor’s office or hospital (for example, COVID-19 or flu),” “It was more convenient than going to the doctor (for example, less travel or wait times),” and “I could include family or other caregivers in my appointment,” with “yes” vs. “no” response options for each item. Similarly, for those who were offered but declined telehealth services, we assessed the deterrents based on the survey question, “Did you choose not to participate in a telehealth visit for any of the following reasons?”, and the deterrent categories, “I preferred to have the appointment(s) in person,” “I was concerned about the privacy of telehealth visits,” and “I thought the telehealth technology would be difficult to use,” with “yes” vs. “no” response options for each item.

### 2.4 Informal caregiving status

The primary independent variable was caregiving status, determined by the following two questions: “Are you currently caring for or making healthcare decisions for someone [a child/children, a spouse/partner, a parent/parents, another family member, a friend or other nonrelative] with a medical, behavioral, disability, or other condition?” Respondents with an affirmative response were then asked a follow-up question, “*Do you provide any of this care professionally as part of a job (for example, as a nurse or professional home health aide)*?” Respondents who answered “yes” to the first and “no” to the second were classified as informal caregivers; all others were considered non-caregivers. A binary variable (yes [informal caregivers] vs. no [non-caregivers]) was created for analyses. To reduce any potential bias, professional caregivers (*n* = 63) were excluded from the analytical sample (Figure 1).

### 2.5 Study covariates

**Figure 2:**
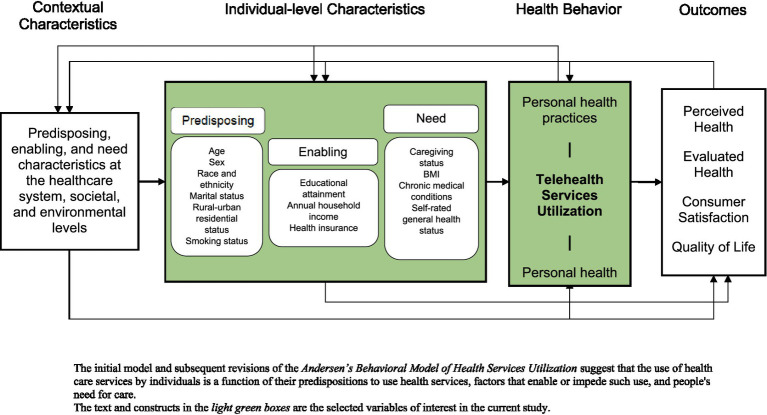
A conceptual framework adopted from Andersen’s behavioral model of health services utilization to assess associations between informal caregiving responsibility and telehealth services utilization among older adults in the United States.

Following Andersen’s Behavioral Model of Health Service Utilization (53, 54), a series of variables were selected and incorporated into the study as covariates. Andersen’s model proposes three core factors that determine, or explain, health service utilization. Those are labeled as predisposing, enabling, and need (need-for-care) factors. The model suggests that individuals’ use of health services is a function of their predispositions to use health services, factors that enable or impede such use, and people’s need for care. The incorporated *predisposing* factors in this study included (age, biological sex, race and ethnicity, marital status, rural–urban residential location, and smoking status). These factors capture sociodemographic and behavioral characteristics that potentially influence the propensity to seek care. *Enabling* (education, income, health insurance) factors generally reflect the logistical and financial resources that facilitate or constrain access to healthcare services. *Need for care* factors represent a person’s subjective need (perceived) (e.g., self-rated general health status) or a clinically assessed condition (evaluated) (e.g., body mass index [BMI], chronic medical conditions [CCs]). Figure 2 depicts a conceptual framework for the current study, adopted from Andersen’s model (53, 54).

### 2.6 Analytical strategy

We began by evaluating data quality and patterns of missingness on the covariates using the PROC MI procedure in SAS. The missing data were minimal and random, allowing for analyses under the missing completely at random (MCAR) assumption. Univariate, bivariate, and multivariable analyses were then performed through a series of steps. All analyses incorporated final sample weights and jackknife replicate weights provided by HINTS to ensure U.S. national-level representativeness and accurate variance estimation. The analytical steps included performing bivariate analysis and reporting sample frequencies with weighted percentages for respondent characteristics. The design-adjusted Rao–Scott chi-square test was used subsequently to investigate bivariate associations between respondents’ predisposing, enabling, and need characteristics and subcategories of telehealth service use. Finally, to assess the associations between informal caregiving status and telehealth utilization, we fit a multivariable multinomial logistic regression by controlling for predisposing, enabling, and need factors. The final computed results included adjusted odds ratios (aORs) and associated 95% confidence intervals (CIs). Our analyses were performed through the SAS 9.4 statistical software program (SAS Institute Inc., Cary, NC, USA), and the statistical significance level was set at *p* ≤ 0.05.

## 3 Results

The analytical sample included 3,354 noninstitutionalized adults aged 50 years and older who reported attempting to schedule medical care in the 12 months prior to the survey. This sample represents an estimated 106.1 million middle-aged and older U.S. adults in 2022. Among respondents, 42.4% reported receiving telehealth services, and 10.3% were offered but did not use telehealth services (Table 1). About 19.8% identified themselves as informal caregivers. Other population level estimates for the predisposing, enabling, and need-for-care characteristics are provided in Table 1.

**Table 1:** Sociodemographic and other characteristics of people who tried to schedule medical care during the past 12 months (ages ≥50 years, HINTS6, the United States, 2022).

Characteristics	Sample (*n* = 3,354)
	Frequency (weighted %)^a^
Telehealth	
Received	1,499 (42.4)
Offered, did not receive	365 (10.3)
Not offered nor received	1,490 (47.3)
Informal caregiver	
Yes	512 (19.8)
No	2,842 (80.2)
Predisposing factors	
Age in years	
50–64	1,493 (56.0)
65–74	1,159 (26.8)
≥75	702 (17.2)
Sex	
Male	1,328 (46.5)
Female	1,958 (53.5)
Race and ethnicity	
Non-Hispanic White	1,971 (66.6)
Non-Hispanic Black	504 (10.1)
Hispanic	412 (10.0)
Non-Hispanic Asian and others	197 (6.2)
Missing or erroneous	270 (7.1)
Marital status	
Married or living as married	1,623 (65.1)
Divorced, widowed, or separated	1,274 (20.9)
Single or never married	381 (14.0)
Rural–urban residential location	
Urban	2,884 (86.5)
Rural	470 (13.5)
Smoking status	
Current	350 (11.6)
Former	1,004 (30.5)
Never	1,911 (57.9)
Enabling factors	
Educational attainment	
Less than high school	211 (5.6)
High school graduate	618 (21.4)
Some college	985 (42.0)
College graduate or more	1,471 (31.0)
Annual household income (US $)	
<20,000	625 (13.9)
20,000-34,999	463 (11.9)
35,000-49,999	462 (11.9)
50,000-74,999	570 (18.3)
≥75,000	1,231 (44.0)
Health insurance coverage	
Yes	3,203 (96.0)
No	127 (4.0)
Need factors	
BMI (kg/m^2^)	
Underweight or normal (≤24.9)	923 (25.6)
Overweight (25–29.9)	1,154 (34.9)
Obese (≥30)	1,208 (38.5)
Chronic medical conditions	
None	692 (35.4)
One	993 (30.4)
≥Two	1,661 (44.2)
Self-rated general health status	
Excellent or very good	1,385 (41.9)
Good	1,274 (39.6)
Fair or poor	661 (18.5)

US, United States; BMI, Body Mass Index (calculated as weight in kilograms divided by height in meters squared).

^a^Frequencies represent sample frequencies while the reported proportions are population-level estimates computed through adjustments for complex design features of the HINTS6 data and the available person weights (*n* = 106.1 million).

Table 2 presents the bivariate associations between telehealth service utilization and respondent characteristics, including caregiving status. Informal caregiving, BMI, number of CCs, and self-rated general health status were significantly associated with telehealth use. Among those who received or were offered but did not receive telehealth, approximately 24.1 and 21.2% were informal caregivers, respectively, compared to only 15.5% among those who neither received nor were offered telehealth (*p* = 0.005). Among telehealth users, 42.6% were obese (BMI ≥ 30 kg/m^2^, *p* = 0.01) 54.1% had two or more CCs (*p* < 0.001), and 22.1% rated their health as fair or poor (*p =* 0.04) (Table 2). Additional descriptive characteristics for two-way associations of caregivers vs. non-caregivers are presented in the Supplementary Table 1.

**Table 2:** Two-way associations of informal caregiving status, other respondents’ predisposing, enabling, and need characteristics, with telehealth services utilization among people who tried to schedule medical care during the past 12 months (ages ≥50 years, HINTS6, *n* = 3,354, the United States, 2022).

	Telehealth			
	Received (*n* = 1,499)	Offered, did not receive (*n* = 365)	Not offered nor received (*n* = 1,490)	
Characteristics	Frequency (weighted %)^a^	Frequency (weighted %)^a^	Frequency (weighted %)^a^	*p*-value^b^
Informal caregiver				
Yes	271 (24.1)	56 (21.2)	185 (15.5)	0.005
No	1,228 (75.9)	309 (78.2)	1,305 (84.5)	
Predisposing factors				
Age in years				
50–64	690 (57.1)	162 (56.3)	641 (55.0)	0.54
65–74	511 (26.4)	133 (30.0)	515 (26.5)	
≥75	298 (16.5)	70 (13.7)	334 (18.5)	
Sex				
Male	563 (43.8)	148 (45.6)	617 (49.1)	0.24
Female	900 (56.2)	208 (54.4)	850 (50.9)	
Race and ethnicity				
Non-Hispanic White	861 (64.2)	213 (64.5)	897 (69.8)	0.37
Non-Hispanic Black	229 (11.4)	53 (8.1)	222 (9.3)	
Hispanic	207 (11.0)	41 (13.5)	164 (8.3)	
Non-Hispanic Asian and others	81 (6.2)	31 (7.7)	85 (5.9)	
Missing or erroneous	121 (7.2)	27 (8.2)	122 (6.7)	
Marital status				
Married or living as married	741 (67.8)	177 (64.2)	705 (62.9)	0.39
Divorced, widowed, or separated	547 (19.4)	142 (22.9)	585 (21.8)	
Single or never married	171 (12.8)	38 (12.9)	172 (15.3)	
Rural–urban residential location				
Urban	1,337 (87.9)	322 (89.2)	1,225 (84.6)	0.06
Rural	162 (12.1)	43 (10.8)	265 (15.4)	
Smoking status				
Current	153 (11.0)	34 (10.5)	163 (12.3)	0.54
Former	465 (32.2)	101 (26.0)	438 (30.0)	
Never	834 (56.8)	221 (63.5)	856 (57.7)	
Enabling factors				
Educational attainment				
Less than high school	80 (4.3)	20 (5.5)	111 (6.7)	0.06
High school graduate	238 (19.3)	59 (21.0)	321 (23.4)	
Some college	424 (40.9)	110 (42.3)	451 (42.8)	
College graduate or more	720 (35.5)	168 (31.2)	583 (27.1)	
Annual household income (US $)				
<20,000	281 (14.2)	56 (13.1)	288 (13.9)	0.33
20,000-34,999	193 (12.1)	49 (11.9)	221 (11.7)	
35,000-49,999	210 (12.7)	43 (10.1)	209 (11.4)	
50,000-74,999	233 (14.8)	70 (21.2)	267 (20.9)	
≥75,000	581 (46.2)	147 (43.7)	503 (42.1)	
Health insurance coverage				
Yes	1,438 (96.7)	351 (96.2)	1,414 (95.2)	0.43
No	50 (3.3)	12 (3.8)	65 (4.8)	
Need factors				
BMI (kg/m^2^)				
Underweight or normal (≤24.9)	404 (27.7)	98 (24.3)	421 (26.0)	0.01
Overweight (25–29.9)	470 (29.7)	141 (43.7)	543 (37.6)	
Obese (≥30)	592 (42.6)	121 (32.0)	495 (36.4)	
Chronic medical conditions				
None	232 (17.4)	81 (32.3)	379 (31.0)	<0.001
One	394 (28.5)	102 (28.6)	497 (32.5)	
≥Two	868 (54.1)	182 (39.1)	611 (36.5)	
Self-rated general health status				
Excellent or very good	554 (38.3)	164 (43.8)	667 (44.8)	0.04
Good	587 (39.6)	137 (41.4)	550 (39.2)	
Fair or poor	338 (22.1)	62 (14.8)	261 (16.0)	

US, United States; BMI, Body Mass Index (calculated as weight in kilograms divided by height in meters squared).

^a^Frequencies represent sample frequencies while the reported proportions are population-level estimates computed through adjustments for complex design features of the HINTS6 data and the available person weights (*n* = 106.1 million).

^b^Design-adjusted Rao-Scott Chi-Square test.

Table 3 displays results from the multivariable multinomial logistic regression model. Compared with non-caregivers, informal caregivers had significantly higher odds of receiving telehealth services (aOR = 1.78; 95% CI: 1.31–2.40). Other significant predictors of telehealth service utilization among the sampled general population were a series of characteristics, including age, race/ethnicity, marital status, educational attainment, and number of CCs. Compared to those aged 50–64, those 75 + years old (aOR = 0.71; 95% CI: 0.51–0.99) had lower odds of receiving telehealth. Hispanics were more likely to receive telehealth compared to non-Hispanic white people (aOR = 1.56; 95% CI: 1.03–2.36). Further, those who were married or living as married (aOR = 1.55; 95% CI: 1.01, 2.37, vs. single or never married) and those with a college degree or more (aOR = 2.52; 95% CI: 1.37, 4.65, vs. less than high school) were more likely to receive telehealth services compared to their counterparts. Lastly, respondents with one CC (aOR = 1.77; 95% CI: 1.18–2.66) and those with two or more CCs (aOR = 3.24; 95% CI: 2.14–4.89) had substantially higher odds of receiving telehealth services than those with no CCs.

**Table 3:** Multivariable multinomial logistic regressions for the associations between informal caregiving status and telehealth services utilization (ages, ≥50 years, HINTS6, *n* = 3,354, the United States, 2022).

	Telehealth	
	Received vs. not offered nor received	Offered, did not receive vs. not offered nor received
Characteristics	Adjusted OR (95% CI)	Adjusted OR (95% CI)
Informal caregiver		
Yes	1.78 (1.31, 2.40)^c^	1.44 (0.78, 2.64)
No	Reference	Reference
Predisposing factors		
Age in years		
50–64	Reference	Reference
65–74	0.80 (0.59, 1.09)	1.05 (0.66, 1.68)
≥75	0.71 (0.51, 0.99)^a^	0.68 (0.38, 1.22)
Sex		
Male	Reference	Reference
Female	1.25 (0.93, 1.67)	1.14 (0.77, 1.69)
Race and ethnicity		
Non-Hispanic White	Reference	Reference
Non-Hispanic Black	1.36 (0.87, 2.12)	0.95 (0.57, 1.58)
Hispanic	1.56 (1.03, 2.36)^a^	1.70 (0.73, 3.92)
Non-Hispanic Asian and others	1.29 (0.77, 2.15)	1.45 (0.76, 2.77)
Missing or erroneous	1.12 (0.69, 1.83)	1.34 (0.55, 3.29)
Marital status		
Married or living as married	1.55 (1.01, 2.37)^a^	1.15 (0.59, 2.25)
Divorced, widowed, or separated	1.22 (0.81, 1.83)	1.29 (0.65, 2.57)
Single or never married	Reference	Reference
Rural–urban residential location		
Urban	Reference	Reference
Rural	0.74 (0.51, 1.08)	0.69 (0.43, 1.10)
Smoking status		
Current	Reference	Reference
Former	1.26 (0.78, 2.02)	1.04 (0.47, 2.30)
Never	1.06 (0.68, 1.67)	1.25 (0.59, 2.65)
Enabling factors		
Educational attainment		
Less than high school	Reference	Reference
High school graduate	1.35 (0.73, 2.50)	1.02 (0.41, 2.52)
Some college	1.68 (0.93, 3.05)	1.13 (0.56, 2.30)
College graduate or more	2.52 (1.37, 4.65)^b^	1.28 (0.64, 2.56)
Annual household income (US $)		
<20,000	Reference	Reference
20,000-34,999	1.07 (0.60, 1.89)	1.28 (0.60, 2.71)
35,000-49,999	1.04 (0.64, 1.68)	1.06 (0.49, 2.29)
50,000-74,999	0.68 (0.39, 1.21)	1.18 (0.59, 2.36)
≥75,000	0.93 (0.52, 1.67)	1.16 (0.58, 2.31)
Health insurance coverage		
Yes	Reference	Reference
No	0.67 (0.30, 1.50)	0.88 (0.27, 2.90)
Need factors		
BMI (kg/m^2^)		
Underweight or normal (≤24.9)	Reference	Reference
Overweight (25–29.9)	0.66 (0.48, 1.03)	1.08 (0.61, 1.91)
Obese (≥30)	0.80 (0.57, 1.11)	0.81 (0.47, 1.40)
Chronic medical conditions		
None	Reference	Reference
One	1.77 (1.18, 2.66)^b^	0.95 (0.58, 1.57)
≥Two	3.24 (2.14, 4.89)^c^	1.24 (0.75, 2.05)
Self-rated general health status		
Excellent or very good	Reference	Reference
Good	1.14 (0.80, 1.63)	1.21 (0.72, 2.03)
Fair or poor	1.39 (0.96, 2.03)	1.22 (0.71, 2.09)

OR, Odds Ratio; CI, Confidence Interval; US, United States; BMI, Body Mass Index (calculated as weight in kilograms divided by height in meters squared).

^a^*p* < 0.05; ^b^*p* < 0.01; ^c^*p* < 0.001.

**Figure 3:**
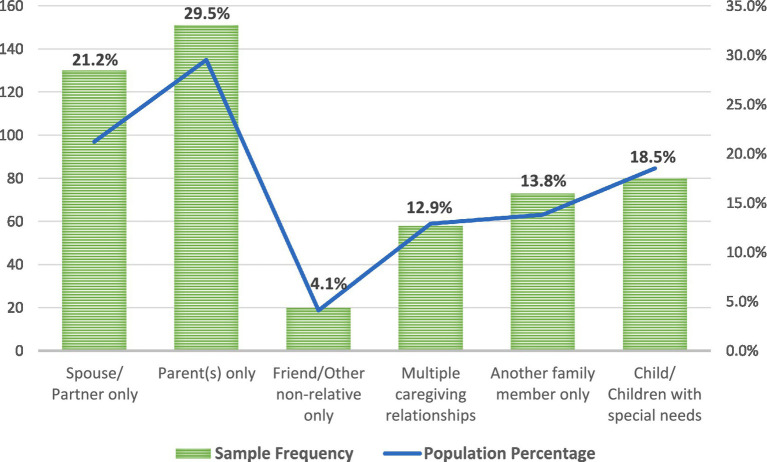
Distribution of caregiving relationships among older informal caregivers (*n* = 512, ages ≥50 years, HINTS6, the United States, 2022).

A higher proportion of informal caregivers reported providing care for parent(s) (29.5%), followed by a spouse/partner (21.2%), and a child or children with special needs (18.5%) (Figure 3). No significant differences were found between caregivers and non-caregivers in the modality of telehealth used. Among caregivers, the most common mode was video-only (52.1%), followed by phone calls (26.4%) and combined video and phone calls (21.5%). Caregivers were more frequent than non-caregivers to cite convenience (66.4% vs. 56.2%, *p* = 0.036) and the ability to involve family members or other caregivers in the appointment (30.8% vs. 19.0%, *p* = 0.037) as key reasons for using telehealth. Conversely, they were less frequently using telehealth to seek advice on whether in-person care was needed (19.8% vs. 28.8%, *p* = 0.04) (Table 4).

**Table 4:** Modes and motivators of telehealth services utilization (informal caregivers vs. non-caregivers) during the COVID-19 pandemic (ages, ≥50 years, HINTS 6, the United States, 2022).

	Informal caregiver		
	Yes (*n* = 271)	No (*n* = 1,228)	
	Frequency (weighted %)^a^	Frequency (weighted %)^a^	*p*-value^b^
Mode of telehealth usage			
Mode of telehealth visit			
Video only	117 (52.1)	501 (41.8)	0.10
Phone call (voice only with no video)	87 (26.4)	485 (36.9)	
Both video and phone call	67 (21.5)	242 (21.3)	
Motivators of telehealth usage			
The health care provider recommended or required the visit use telehealth			
Yes	181 (65.1)	811 (74.0)	0.10
No	71 (34.9)	309 (26.0)	
I wanted advice about whether I needed in-person medical care			
Yes	56 (19.8)	278 (28.8)	0.04
No	194 (80.2)	835 (71.2)	
I wanted to avoid possible infection at the doctor’s office or hospital (for example, COVID-19 or flu)			
Yes	124 (53.2)	518 (46.9)	0.24
No	129 (46.8)	603 (53.1)	
It was more convenient than going to the doctor (for example, less travel or wait times)			
Yes	151 (66.4)	624 (56.2)	0.036
No	103 (33.6)	491 (43.8)	
I could include family or other caregivers in my appointment			
Yes	65 (30.8)	195 (19.0)	0.037
No	187 (69.2)	917 (81.0)	

^a^Frequencies represent sample frequencies while the reported proportions are population-level estimates computed through adjustments for complex design features of the HINTS6 data and the available person weights.

^b^Design-adjusted Rao-Scott Chi-Square Test.

**Figure 4:**
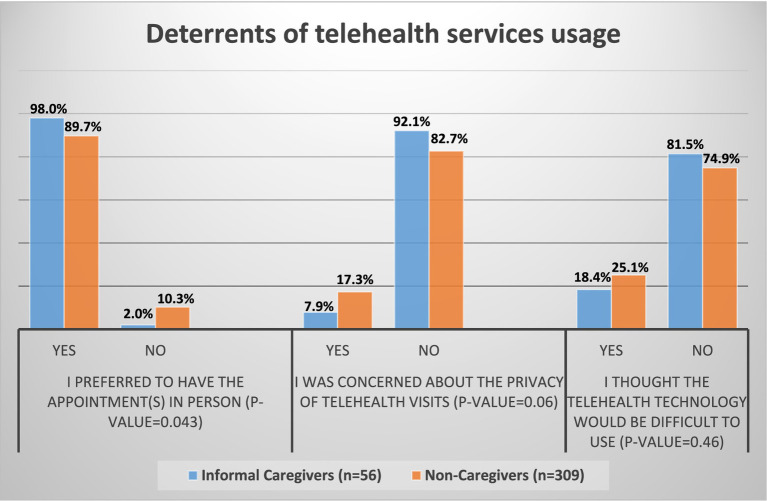
Deterrents of telehealth services usage among individuals who tried to schedule medical care and were offered but did not use telehealth services during the past 12 months (*n* = 365, ages ≥50 years, HINTS 6, the United States, 2022).

Among caregivers who were offered but did not use telehealth, 98.0% (vs. 89.7% non-caregivers) preferred in-person appointments (*p* = 0.043). Further, privacy concerns were less prevalent among caregivers (7.9%) compared to non-caregivers (17.3%) (*p* = 0.06) (Figure 4).

## 4 Discussion

This study used nationally representative data from the U.S. to examine the association between informal caregiving status and telehealth service utilization among middle to older-age adults (ages ≥50 years) during the COVID-19 pandemic. It also explored telehealth use modalities, motivators, and deterrents among informal caregivers. Our findings indicate that informal caregivers were significantly more likely than non-caregivers to receive telehealth services. Additionally, caregivers demonstrated distinct usage patterns, driven by convenience and opportunities for family participation, while also expressing higher preference for in-person care when not using telehealth. These findings suggest that caregiving status was an independent correlate of telehealth receipt among middle-aged and older adults during the pandemic. They also underscore the need for telehealth policies and interventions tailored to the unique needs of informal caregivers, an essential but often overlooked population in the healthcare system.

Informal caregivers reported higher rates of receiving telehealth services compared to non-caregivers and had almost twice the odds of receiving telehealth services. These findings highlight the pertinence of telehealth as a potentially valuable tool for informal caregivers in two keyways. First, increased likelihood of being offered and receiving telehealth services may reflect healthcare providers’ recognition of caregivers’ needs for flexible care options, as well as caregivers’ willingness to engage with remote care modalities (8). Second, increased utilization of telehealth among informal caregivers may reflect their greater perceived or actual need for healthcare, particularly during the pandemic. This aligns with existing literature that highlights caregivers’ fair to poor health status and increased healthcare needs due to stress, mental health issues, comorbid conditions, and care coordination demands (55–57). Given that caregivers often manage both their own health and that of their care recipients, our results reinforce the importance of integrating caregiver-specific considerations into telehealth service planning and delivery.

Further, our findings provide valuable insights into the modalities and motivators of telehealth use among informal caregivers and non-caregivers. In this study, telehealth modalities included telehealth by video only (videoconferencing with both audio and visual communication), by phone call (voice only with no video), and combined video and phone call use. Although modality distribution did not differ significantly between caregivers and non-caregivers, the pattern suggests greater video-only use among this group of caregivers (52.1% vs. 41.8%), with lower reliance on phone call (26.4% vs. 36.9%). Even without statistical significance, this pattern aligns with a preference for more interactive visits when care coordination and shared decision-making are salient (58, 59). The finding suggests a growing comfort with visual communication technologies among mature informal caregivers. Further, this trend is consistent with recent reviews noting increased acceptance and satisfaction with videoconferencing among caregivers (59), although some telehealth interventions continue to rely heavily on telephone-based delivery (58). Together, these findings highlight the need for multi-modal telehealth platforms that account for varying access to technology and user preferences (8).

Informal caregivers more frequently used telehealth for reasons of convenience, with 66.4% of caregivers indicating that telehealth was more convenient than in-person visits, compared to 56.2% of non-caregivers. This finding suggests that telehealth may be particularly appealing to middle-aged and older informal caregivers, as it reduces the time and effort required to travel or wait for in-person appointments. By offering a more flexible and accessible healthcare delivery option, telehealth can provide critical support for individuals managing caregiving responsibilities, as highlighted in prior studies (8, 60). Additionally, a higher proportion of informal caregivers in our study reported that telehealth allowed them to include family members or other caregivers in the appointment (30.8% vs. 19.0%). This underscores the potential of telehealth to facilitate shared decision-making and provide a collaborative care experience, which is crucial for caregivers who often have to manage multiple health-related responsibilities for those they care for (61, 62). Conversely, informal caregivers used telehealth for advice about whether in-person medical care was needed (19.8% vs. 28.8%) less frequently than non-caregivers, suggesting that informal caregivers typically engage in telehealth with more specific or ongoing care needs, rather than for diagnostic uncertainty. Altogether, this profile suggests informal caregivers employ telehealth deliberately for ongoing management and coordination needs rather than for diagnostic triage alone (60–62). These findings emphasize the importance of tailoring telehealth services to address the practical and relational needs of informal caregivers while ensuring support for diverse usage motivations.

Deterrents of telehealth use also diverged. Among those who were offered but did not use telehealth, preference for in-person care was nearly universal among informal caregivers (98.0% vs. 89.7%, *p* = 0.043). Further, privacy concerns trended lower in caregivers (7.9% vs. 17.3%, *p* = 0.06). Contrary to assumptions that informal caregivers face greater digital barriers, these data imply that when caregivers decline telehealth, the dominant reason is clinical preference for in-person contact, not privacy anxieties or usability obstacles (43, 63–65).

Beyond caregiving status, several covariates, including age, race/ethnicity, marital status, educational attainment, and number of chronic conditions, were significantly associated with higher or lower odds of telehealth use, supporting the robustness of our regression model. Adults aged ≥75 years had lower odds of receiving telehealth compared to those aged 50–64, likely reflecting barriers such as lower digital health literacy, limited broadband access, sensory or cognitive limitations, and a preference for in-person care (19, 66). However, it is important to also consider that lower telehealth use among the oldest adults may not solely represent a gap, or access deficit, but may reflect patient preferences for in-person care.

Moreover, in contrast to some prior studies reporting lower telehealth use among Hispanic populations (28, 35, 66), our findings showed higher odds of receiving telehealth among Hispanics compared to non-Hispanic white people, which may reflect improved access through culturally and linguistically tailored services or evolving patterns of healthcare during and following the COVID-19 pandemic ((67, 68)). Higher educational attainment, especially among those with a college degree or higher, was also associated with greater odds of receiving telehealth, consistent with prior evidence (69, 70) and possibly reflecting greater digital and health literacy and comfort navigating online systems. Similarly, having one or more chronic conditions was associated with higher odds of receiving telehealth, consistent with increased healthcare needs, more frequent interactions with the healthcare system, and greater opportunity to adopt telehealth modalities (69, 70). Together, these findings highlight that telehealth utilization is shaped by intersecting predisposing, enabling, and need factors, and suggest the importance of continued targeted efforts to reduce persistent inequities in telehealth access.

### 4.1 Limitations

This study has a few limitations that warrant caution. First, its cross-sectional design limits the ability to infer causality between caregiving status and telehealth utilization. Second, the data were self-reported, which may introduce biases such as recall bias or social desirability bias. Third, while the study utilized a nationally representative sample of U.S. informal caregivers, findings may not be generalizable to populations in other countries with different healthcare systems, cultural norms, or telehealth infrastructure. Fourth, unmeasured confounding variables could influence the observed associations. Notably, the dataset lacked variables addressing key contextual factors, such as the availability of in-person healthcare services, quality or satisfaction with telehealth services, or technological barriers that could affect telehealth access—such as internet connectivity, device availability, or digital literacy. Fifth, the HINTS dataset did not distinguish whether telehealth services were utilized by caregivers or intended for their care recipients, nor did it provide information about the type or setting of telehealth services used (e.g., primary vs. specialty care). Lastly, although HINTS6 data were collected in 2022, respondents reported healthcare experiences and telehealth offer and/or use over the prior 12 months, capturing a period that spans both earlier and later phases of the COVID-19 pandemic. As such, telehealth utilization patterns observed in this study may reflect lingering effects of earlier disruptions to in-person care. Acknowledging these limitations can inform future research efforts aimed at generating more comprehensive and causal insights into the relationship between caregiving responsibilities and telehealth use.

## 5 Conclusion

Informal caregivers aged 50 or older were significantly more likely than non-caregivers to receive telehealth services during the COVID-19 pandemic. Caregivers prioritized convenience and involving family/other caregivers during visits, and when telehealth was declined, the predominant deterrent was a preference for in-person care, not privacy or usability concerns. These findings have important implications for public health practice and healthcare policy, particularly as healthcare systems expand telehealth offerings and seek better support for caregivers. To meet caregivers’ unique needs, healthcare systems could design telehealth platforms that prioritize convenience and allow for family participation. Telehealth strategies that (1) support multi-participant visits, (2) optimize scheduling and access, and (3) provide coordinated after-visit information are well-positioned to reduce caregiver burden and enhance care continuity among caregivers. These features are especially valuable in managing chronic conditions, older adults care, and post-hospital recovery, areas where informal caregivers are essential. The results also highlight critical health and policy considerations for informal caregivers, who compose a critical share of the caregiving workforce and often manage significant personal health needs alongside caregiving duties. Despite their higher burden of chronic conditions and higher likelihood of needing ongoing care themselves, the oldest (aged 75 + years) adults in this study exhibited lower odds of receiving telehealth services. This underscores the need for targeted support for this group to help address persistent gaps.

Additionally, more comprehensive standards and regulations may be necessary to ensure robust privacy and security protections for telehealth users (63). Reimbursement and coverage policies should also recognize the vital role of informal caregivers and promote flexible, inclusive models that accommodate their responsibilities (56). By aligning telehealth systems with caregivers’ realities, we can strengthen care coordination, reduce caregiver burden, and improve outcomes for caregivers and care recipients alike.

## Data Availability

Publicly available datasets were analyzed in this study. This data can be found here: the data analyzed for the current study were obtained from the National Cancer Institute’s Health Information National Trends Survey; publicly available to access and download at: https://hints.cancer.gov/.
