# Supplementary material for: Telehealth services utilization among middle-aged and older informal caregivers: evidence from national-level data in the United States
Source: Front Public Health. 2026 Jun 16;14:1859507. doi: 10.3389/fpubh.2026.1859507 (PMC13315035; doi:10.3389/fpubh.2026.1859507)
Supplement: Supplementary file 1 [file Table_1.DOCX]

**Supplementary Table 1.** Two-way associations between informal caregiving status and predisposing, enabling, and need characteristics of people who tried to schedule medical care during the past 12 months (ages ≥50 years, HINTS6, n=3,354, the United States, 2022).

|  |  |  |  |
| --- | --- | --- | --- |
|  | Informal Caregiver | |  |
|  | Yes (n=512) | No (n=2,842) |  |
| Characteristics | Frequency (weighted %)^a^ | Frequency (weighted %)^a^ | P-value^b^ |
| **Predisposing factors** |  |  |  |
| Age in years |  |  |  |
| 50-64 | 268 (64.7) | 1,225 (53.9) | 0.003 |
| 65-74 | 162 (22.3) | 997 (27.9) |  |
| ≥75 | 82 (13.0) | 620 (18.2) |  |
| Sex |  |  |  |
| Male | 169 (41.5) | 1,159 (47.8) | 0.14 |
| Female | 330 (58.5) | 1,628 (52.2) |  |
| Race and ethnicity |  |  |  |
| Non-Hispanic White | 289 (66.2) | 1,682 (66.7) | 0.29 |
| Non-Hispanic Black | 70 (8.7) | 434 (10.4) |  |
| Hispanic | 73 (12.5) | 339 (9.4) |  |
| Non-Hispanic Asian and others | 41 (7.6) | 156 (5.9) |  |
| Missing or erroneous | 39 (5.0) | 231 (7.6) |  |
| Marital status |  |  |  |
| Married or living as married | 327 (76.5) | 1,296 (62.2) | <0.001 |
| Divorced, widowed, or separated | 129 (14.3) | 1,145 (22.6) |  |
| Single or never married | 44 (9.2) | 337 (15.2) |  |
| Rural-urban residential location |  |  |  |
| Urban | 449 (87.1) | 2,435 (86.3) | 0.70 |
| Rural | 63 (12.9) | 407 (13.7) |  |
| Smoking status |  |  |  |
| Current | 53 (12.0) | 297 (11.4) | 0.04 |
| Former | 125 (22.9) | 879 (32.5) |  |
| Never | 322 (65.1) | 1,589 (56.1) |  |
| **Enabling factors** |  |  |  |
| Educational attainment |  |  |  |
| Less than high school | 24 (4.2) | 187 (5.9) | 0.12 |
| High school graduate | 80 (18.3) | 538 (22.2) |  |
| Some college | 148 (40.6) | 837 (42.3) |  |
| College graduate or more | 249 (36.9) | 1,222 (29.6) |  |
| Annual household income (US $) |  |  |  |
| <20,000 | 73 (10.6) | 552 (14.7) | 0.11 |
| 20,000-34,999 | 67 (12.7) | 396 (11.7) |  |
| 35,000-49,999 | 65 (11.3) | 397 (12.0) |  |
| 50,000-74,999 | 90 (14.9) | 480 (19.2) |  |
| ≥75,000 | 216 (50.5) | 1,015 (42.4) |  |
| Health insurance coverage |  |  |  |
| Yes | 492 (95.6) | 2,711 (96.0) | 0.74 |
| No | 20 (4.4) | 107 (4.0) |  |
| **Need factors** |  |  |  |
| BMI (kg/m^2^) |  |  |  |
| Underweight or normal (≤24.9) | 137 (23.5) | 786 (27.3) | 0.45 |
| Overweight (25-29.9) | 169 (36.0) | 985 (34.6) |  |
| Obese (≥30) | 199 (40.5) | 1,009 (38.1) |  |
| Chronic medical conditions |  |  |  |
| None | 108 (31.9) | 584 (23.8) | 0.11 |
| One | 160 (28.4) | 833 (30.9) |  |
| ≥Two | 244 (39.7) | 1,417 (45.3) |  |
| Self-rated general health status |  |  |  |
| Excellent or very good | 209 (44.2) | 1,176 (41.4) | 0.63 |
| Good | 203 (36.6) | 1,071 (40.3) |  |
| Fair or poor | 96 (19.2) | 565 (18.3) |  |
| Abbreviations: US, United States; BMI, Body Mass Index (calculated as weight in kilograms divided by height in meters squared). | | | |
| ^a^ Frequencies represent sample frequencies while the reported proportions are population-level estimates computed through adjustments for complex design features of the HINTS6 data and the available person weights (N=106.1 million). | | | |
| ^b^ Design-Adjusted Rao-Scott Chi-Square Test | |  |  |
